# PB1 as a potential target for increasing the breadth of T-cell mediated immunity to Influenza A

**DOI:** 10.1038/srep35033

**Published:** 2016-10-07

**Authors:** Ida E. M. Uddbäck, Maria A. Steffensen, Sara R. Pedersen, Loulieta Nazerai, Allan R. Thomsen, Jan P. Christensen

**Affiliations:** 1 Department of Immunology and Microbiology, University of Copenhagen, Copenhagen, Denmark

## Abstract

Recently, we showed that combined intranasal and subcutaneous immunization with a non-replicating adenoviral vector expressing NP of influenza A, strain PR8, induced long-standing protection against a range of influenza A viruses. However, H-2^b^ mice challenged with an influenza A strain mutated in the dominant NP_366_ epitope were not efficiently protected. To address this problem, we envision the use of a cocktail of adenovectors targeting different internal proteins of influenza A virus. Consequently, we investigated the possibility of using PB1 as a target for an adenovector-based vaccine against influenza A. Our results showed that PB1 is not as immunogenic as the NP protein. However, by tethering PB1 to the murine invariant chain we were able to circumvent this problem and raise quite high numbers of PB1-specific CD8^+^ T cells in the circulation. Nevertheless, mice immunized against PB1 were not as efficiently protected against influenza A challenge as similarly NP-vaccinated animals. The reason for this is not a difference in the quality of the primed cells, nor in functional avidity. However, under similar conditions of immunization fewer PB1-specific cells were recruited to the airways, and surface expression of the dominant PB1 peptide, PB1_703_, was less stable than in the case of NP_366_.

Influenza A virus is a very common respiratory virus causing 3–5 million cases of severe disease each year worldwide^1^. Symptoms include fever, muscle pain, sore throat and coughing. Most people recover from the infection but between 250.000–500.000 humans succumb to the disease annually^1^. Existing antibody based vaccines target the surface proteins HA and NA, which is problematic due to their high degree of antigenic variation^2^. Point mutations (antigenic drift) and genetic reassortment (antigenic shift) change these surface glycoproteins, so that existing antibodies rapidly become of limited protective value. Due to this, ongoing global surveillance and typically the production of a new vaccine combination is required for each winter season. Not only is this an expensive process, but if the predictions turn out to be wrong, then the consequences may be devastating. Further, this vaccine strategy does not work in relation to new pandemic strains, since the antigenic composition is stochastic and unpredictable. Consequently, there is a need for the development of a new vaccine strategy.

In contrast to antibody mediated immunity, CD8^+^ T-cell immunity also targets the conserved internal proteins of influenza. Several studies, in both mice and man, have identified CD8^+^ T cells to be of major importance for clearance of influenza virus infections, and CD8^+^ memory T cells can remain for at least a couple of years after the infection is cleared^3^,^4^,^5^,^6^,^7^. As CD8^+^ T cells target infected cells, not cell-free virus, T cells from influenza primed mice do not prevent infection but causes earlier clearance from the lungs and hence protects the mice from otherwise lethal disease^8^. T-cell mediated immunity can therefore provide a basic protection against influenza A, important in certain times of need, such as during a pandemic, when the majority of the population lacks antibody mediated protection. If we can induce long lasting CD8^+^ T-cell immunity, the population would also be protected from severe disease and the deadly consequences of seasonal flu. We have previously described a replication-deficient adenovirus vector encoding the influenza A nucleoprotein (AdNP) with the capacity to induce a long-standing CD8^+^ memory T-cell response against influenza infection^9^. In the same study, we also demonstrated that the protection, induced by the vaccine, is exerted predominantly by CD8^+^ T cells. Adenovirus serotype 5 used in this study has been shown to induce high quality CD8^+^ memory T-cell when used as a vaccine vector^10^. The fact that adenovirus naturally infects the airways and therefore may induce homing of immune cells to the lungs also speaks in its favor for usage as a vector in an influenza vaccine. Several studies have used adenovirus vectors encoding influenza genes HA, M1 or NP to vaccinate against influenza with varying success^11^,^12^,^13^. Even though the internal proteins are highly conserved between influenza A strains, variations do exist which could cause the virus to escape CD8^+^ T-cell mediated immunity. To circumvent this problem, we propose a vaccine-cocktail, targeting several of the internal proteins of the influenza virus, creating a vaccine able to protect against challenge with a broad variety of strains. PB1 is highly conserved^8^ and has previously been sparsely studied as a vaccine target. A DNA vaccine expressing PB1 has shown promise of protective capacity. However, immunization had to be performed trice to boost the immune response, and mice were only challenged with low doses^14^. One limitation in this context could be a low intrinsic immunogenicity of PB1. However, we have recently found that CD8^+^ T-cell responses directed towards weak antigens may be markedly augmented by expressing the antigen linked to the invariant chain (Ii); thus tethering of the antigen to Ii increases the number of epitope/MHC class I complexes on the cell surface and probably through this mechanism accelerates, augments and prolongs the antigen-specific CD8^+^ T-cell responses particularly against epitopes of low/intermediate immunogenicity^15^,^16^. The aim of this study is therefore to investigate the protective capacities of a replication deficient adenovirus encoding PB1 with and without Ii chain (AdIiPB1 and AdPB1, respectively). H-2^b^ mice vaccinated both locally and systemically with AdIiPB1 were significantly protected up to 60 days post vaccination compared to non-vaccinated mice. However, despite high numbers of virus-specific CD8^+^ T cells in the circulation, the protection never matched that following AdNP vaccination. Part of the reason for this appears to be a less stable surface expression of the major PB1 epitope (PB1_703_), leading to a reduced capacity of PB1-specific cells to kill target cells expressing this epitope compared to the killing of NP_366_ expressing cells by NP_366_-specific CD8^+^ T cells. Consequently, a vaccine targeting PB1 is not sufficient as a stand-alone vaccine, even when tethered to Ii, but PB1 may be included in a combination with other internal genes of influenza.

## Results

### Adenovirus encoding PB1 linked to invariant chain induces high number of antigen-specific CD8^+^ T cells

**Figure 1:**
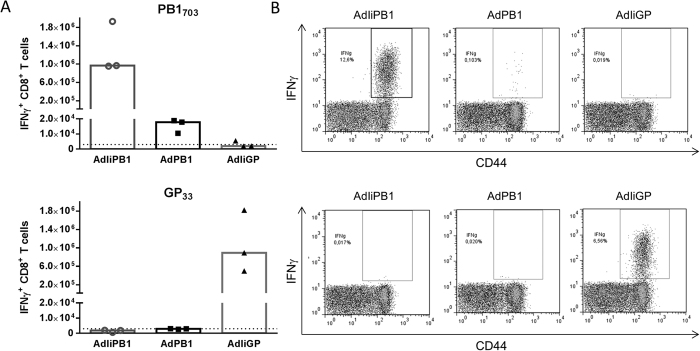
Systemic vaccination with AdIiPB1 induces a potent PB1-specific CD8^+^ T-cell response. (**A**) C57BL/6 mice were vaccinated subcutaneously (s.c.) in the foot pad with 2 × 10^7^ of PFU AdIiPB1 or AdPB1 and splenocytes were isolated 14 days later. Antigen-specific CD8^+^ T cells were enumerated using ICS and flow cytometry after peptide stimulation. Each dot represents one animal and bars represent medians. Dotted line represents background. Splenocytes from mice vaccinated with an adenovector expressing the irrelevant glycoprotein of lymphocytic choriomeningitis virus (AdIiGP) were included to document the antigen-specificity of CD8^+^ T cells induced by Ii-tethered antigen. (**B**) Representative dot plots of gated CD8^+^ T cells from each group. In both cases results in the upper row represent *ex vivo* stimulation with PB1_703_, and in the lower row with GP_33_.

**Figure 2:**
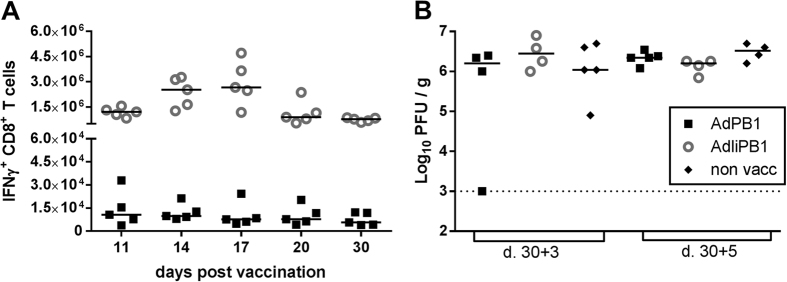
Despite the induction of a potent CD8^+^ T-cell response, AdIiPB1 does not protect against influenza challenge. (**A**) C57BL/6 mice were vaccinated s.c. in the foot pad with 2 × 10^7^ of PFU AdIiPB1 or AdPB1 and splenocytes was isolated 11, 14, 17, 20 and 30 days later. Antigen-specific CD8^+^ T cells were enumerated using flow analysis after peptide stimulation. Each dot represents one animal and bars represent medians. (**B**) C57BL/6 mice were vaccinated s.c. with 2 × 10^7^ of PFU AdIiPB1 or AdPB1 and challenged intranasal (i.n.) 30 days later with PR8 virus. Lungs were isolated 3 and 5 days post challenge and viral titer was measured using MDCK plaque assay. Each dot represents one animal and lines represent medians. Dotted line represents the detection limit of the MDCK plaque assay.

Adenovirus vaccines encoding PB1 with or without invariant chain (AdPB1 or AdIiPB1) were used to vaccinate mice s.c. in the foot pad; an adenovector expressing invariant chain combined with an irrelevant antigen (the glycoprotein of lymphocytic choriomeningitis virus, AdIiGP) was used as control. Fourteen days after vaccination, and following *ex vivo* stimulation with PB1_703_ peptide, numbers of antigen-specific, IFN-γ^+^ CD8^+^ T cells from the spleen were enumerated through intracellular cytokine staining and flow cytometry. A significantly higher numbers of PB1-specific CD8^+^ T cells were detected in animals vaccinated with the construct expressing PB1 tethered to invariant chain, AdIiPB1, compared to the untethered construct (Fig. 1A+B). Very few PB1-specific CD8^+^ T cells were detected in mice vaccinated with AdPB1, and, notably, tethering to invariant chain did not influence the antigen-specificity of the elicited CD8^+^ T-cell response. In mice vaccinated with AdIiPB1, the response peaked between day 14 and 17 after vaccination (Fig. 2A), and decreased until day 20, where after the number of cells remained stable up to 30 days post vaccination. Despite the high numbers of PB1 specific cells elicited in AdIiPB1 vaccinated mice, we found little or no protection when vaccinated mice were challenged after 30 days with homologous influenza virus PR8, neither 3 nor 5 days post challenge (Fig. 2B).

### Vaccination systemically and locally with AdIiPB1 increases capacity to control influenza challenge

Previous studies have shown that i.n. vaccination increases the number of antigen specific CD8^+^ T cells in the lungs and can therefore provide better protection against a respiratory infection^17^. Even though we observed no protection from s.c. vaccination with neither AdIiPB1 nor AdPB1, we hypothesized that due to the high number of antigen specific CD8^+^ T cells in the spleen induced by AdIiPB1, AdIiPB1 could still induce significant protection if the primed cells were effectively recruited to the lungs. To test this theory, C57BL/6 mice were vaccinated systemically (s.c.), locally (i.n.) or through both routes (s.c. + i.n.) and then challenged with PR8 virus 30 days later. Viral titers were measured in the lungs 3 and 5 days post challenge.

**Figure 3:**
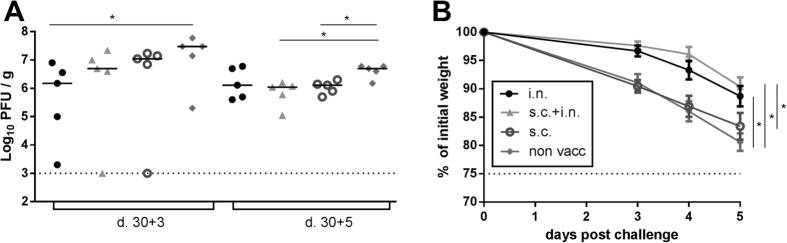
Clinical consequences of local and systemic vaccination. C57BL/6 mice were vaccinated with 2 × 10^7^ of PFU AdIiPB1 s.c., i.n. or both s.c. and i.n. 30 days after vaccination, mice were challenged with PR8 virus. (**A**) 3 and 5 days post challenge, lungs were isolated and viral titer determined using MDCK plaque assay. Each dot represents one animal and lines represent medians. The dotted line represents the detection limit of the assay. (**B**) Percentage weight loss of initial weight post challenge. Dots represent mean and bars SEM. Dotted line represents end point for experiment, 75%. *p < 0.05.

Mice that were vaccinated both locally and systemically tended to have lower viral titers in their lungs compared to unvaccinated controls on day 3 post infection, and on day 5 after challenge a significant reduction was found (Fig. 3A). Unlike most experiments (cf. Fig. 2B), even s.c. vaccinated mice had significantly reduced viral titers on day 5. Mice vaccinated exclusively by the i.n. route were the only mice to have significantly lower lung virus levels compared to unvaccinated mice on day 3 after challenge, and, notably, this difference disappeared when similar mice were analyzed 2 days later; this pattern suggests that the effect of local vaccination per se wanes with time after challenge.

Parallel to the measurements of viral loads, the infection-induced weight loss was recorded after challenge and until the animals were sacrificed (Fig. 3B). Mice vaccinated through both routes displayed less morbidity 3–5 days post challenge compared to mice vaccinated s.c. or unvaccinated mice. Even though the protection of mice vaccinated only i.n. seemed to fade with time, these mice were still clinically protected, displaying a weight loss on days 3–5 post challenge marginally bigger than that of mice vaccinated both locally and systemically. Nevertheless, if both viral loads and weight loss are considered, the overall impression was that combined vaccination provided the most consistent protection.

### Increased number of antigen specific CD8^+^ T cells in the lungs of mice vaccinated both systemically and locally.

**Figure 4:**
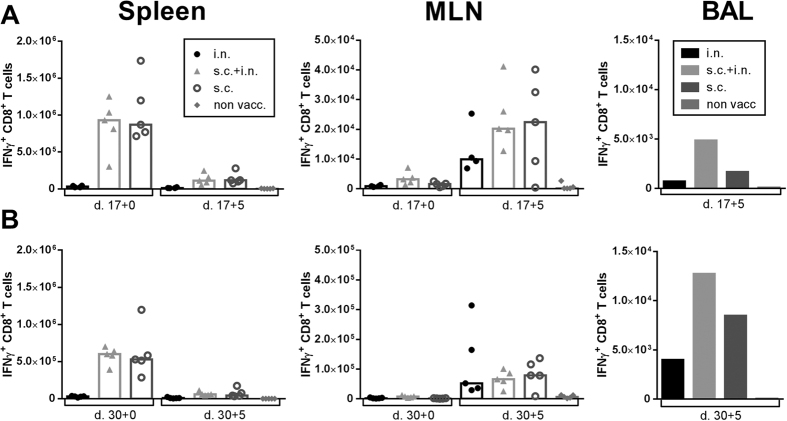
A higher number of antigen specific cells are present in the lungs when mice are vaccinated both systemically and locally. C57BL/6 mice were vaccinated with 2 × 10^7^ of PFU AdIiPB1 s.c., i.n. or both s.c. and i.n. 17 and 30 days after vaccination spleen and MLN were isolated from half of the mice and the number of IFN-γ^+^ CD8^+^ T cells in each organ site was determined through flow analysis after peptide stimulation. The remaining mice were challenged 17 and 30 days post vaccination with PR8 virus and 5 days post challenge spleen, MLN and BAL were isolated. The number of IFN-γ^+^ CD8^+^ T cells in each organ site was determined through flow analysis after peptide stimulation. Each dot represents one animal and bars represent medians (spleen and MLN). In BAL bars represent average number of IFN-γ^+^ CD8^+^ T cells per animal based on a pool from 5 animals.

Knowing that combined local and systemic vaccination could reduce the viral titer in challenged mice, we wanted to investigate how the different vaccination routes impacted the locations of CD8^+^ T cells following viral challenge. C57BL/6 mice were vaccinated i.n., s.c, or s.c and i.n. with AdIiPB1. 17 (Fig. 4A) and 30 days (Fig. 4B) after vaccination, PB1-specific IFNγ^+^ CD8^+^ T cells in the spleen and MLNs were enumerated after peptide stimulation in half of the mice through intracellular staining and flow cytometry. The other half was challenged with PR8 virus and PB1-specific CD8^+^ T cells from the spleen, MLNs and BAL were enumerated after peptide stimulation through flow cytometry 5 days post challenge. As seen in Fig. 3, high numbers of IFNγ^+^ CD8^+^ T cells were detected in the spleen before challenge of mice vaccinated s.c. + i.n. or s.c., and these numbers were markedly reduced 5 days after challenge. In contrast, the number of PB1-specific cells in the MLN increased in all groups post challenge. There was no difference in the number of cells in the MLN between the groups 5 days post challenge. This was found both for mice challenged 17 as well as 30 days post vaccination. Notably, numbers of IFNγ^+^ CD8^+^ T cells in the BAL 5 days post challenge were consistently higher in the mice vaccinated via both routes compared to a single route.

### Systemic and local vaccination with AdliPB1 can induce long-lasting protection

**Figure 5:**
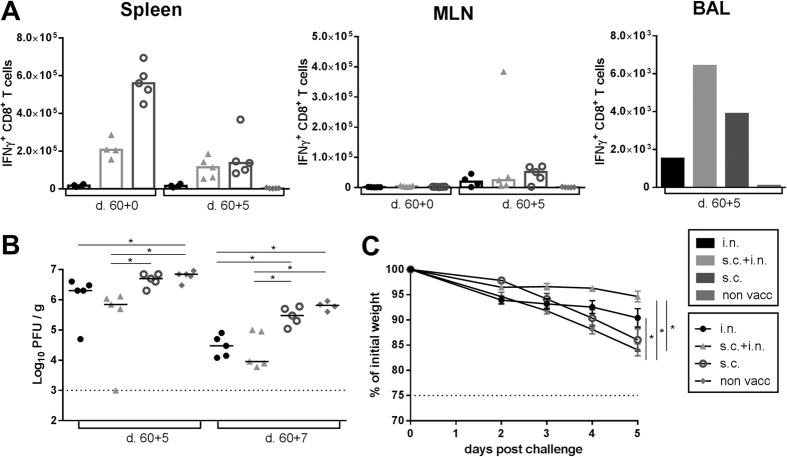
Systemic and local AdIiPB1 vaccination combined induces long lasting protection. (**A**) C57BL/6 mice were vaccinated with 2 × 10^7^ of PFU AdIiPB1 s.c., i.n. or both s.c. and i.n. 60 days post vaccination, spleen and MLN were isolated from half of the mice and the number of IFNγ^+^ CD8^+^ T cells in each organ site was determined though flow analysis after peptide stimulation. The remaining mice were challenged with PR8 virus and 5 days post challenge spleen, MLN and BAL were isolated. The number of IFN-γ^+^ CD8^+^ T cells in each organ site was determined though flow analysis after peptide stimulation. Each dot represents one animal and bars represent medians (spleen and MLN). In BAL bars represent the average number of IFN-γ^+^ CD8^+^ T cells per animal based on a pool from 5 animals. (**B**) C57BL/6 mice were vaccinated with 2 × 10^7^ of PFU AdIiPB1 s.c., i.n. or both s.c. and i.n. 60 days post vaccination mice were challenged with PR8, and 5 and 7 days post challenge lungs were isolated and viral titer determined using MDCK plaque assay. Each dot represents one animal and lines represent medians. The dotted line represents the detection limit of the assay. (**C**) Percentage weight loss of the initial weight in C57BL/6 mice vaccinated and challenged as described in B up until 5 days post challenge. *p < 0.05.

Next, we wanted to examine if the capacity of AdIiPB1 to induce protective CD8^+^ T cells lasted into the memory phase. To this end, C57BL/6 mice were vaccinated s.c., i.n. or both s.c. and i.n. and challenged with PR8 virus 60 days later. Analysis of viral titers in the lungs 5 days post challenge revealed a small, but statistically significant difference between several of the vaccinated groups and unvaccinated controls (Fig. 5B), and the lowest titers were found in mice subjected to the combined vaccination. This pattern was repeated 7 days post challenge. Regarding the virus-induced morbidity, we again followed the weight loss. Similar to what was found on day 30 post vaccination, mice vaccinated both systemically and locally lost significantly less weight compared to mice vaccinated s.c. or non-vaccinated mice (Fig. 5C). Analysis of CD8^+^ T cells isolated from the spleen, MLN and BAL (Fig. 5A) showed similar patterns as on day 17 and 30 (Fig. 4A,B) post vaccination and are also present in almost comparable numbers indicating a good recall response.

### Systemic and local vaccination partially protects mice from succumbing to lethal influenza challenge.

**Figure 6:**
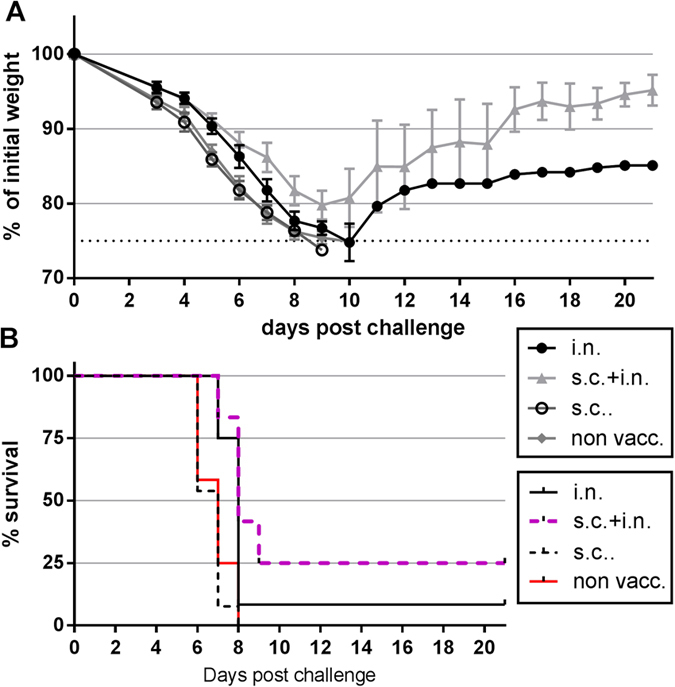
Systemic and local AdIiPB1 vaccination combined decreases morbidity and mortality. C57BL/6 mice were vaccinated with 2 × 10^7^ of PFU AdIiPB1 s.c., i.n. or both s.c. and i.n. 60 days post vaccination mice were challenged with PR8 virus. Percentage weight loss of initial weight (**A**) and survival rate (**B**) was recorded up to 21 days post challenge.

To investigate the morbidity and mortality after challenge of mice vaccinated with AdIiPB1 both i.n. as well as s.c., groups of mice were followed until 21 days after challenge. C57BL/6 mice were vaccinated s.c., i.n. or by both routes, and 60 days later all mice were challenged with a lethal dose of PR8. Mice were weighed daily (Fig. 6A) and survival (Fig. 6B) was recorded. Neither the mice in the control group nor the mice vaccinated s.c. survived past day 8. 25% of the mice vaccinated both s.c. and i.n. survived the infection and displayed no weight loss 21 days post challenge. In the group of mice vaccinated only i.n., a very small proportion survived, and these mice had not regained as much weight 21 days post challenge as had the mice subjected to combined vaccination.

### Vaccination against PB1 does not protect as efficiently as vaccination against NP

**Figure 7:**
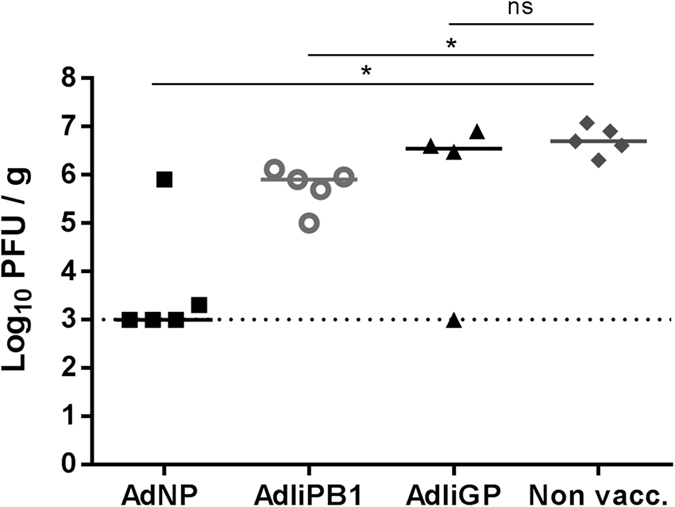
AdIiPB1 vaccination does not match AdNP vaccination with regard to antiviral protection. C57BL/6 mice were vaccinated i.n. plus s.c. with one of the following adenovector constructs: AdIiPB1, AdNP or AdIiGP for control. Thirty days later these mice and a group of naïve controls were challenged i.n. with PR8. Five days later lungs were isolated and viral titer determined using MDCK plaque assay. Each dot represents one animal and lines represent medians. The dotted line represents the detection limit of the assay.

The antiviral protection presented above (and associated with PB1vaccination) appear not to match that previously published for NP vaccination^9^, suggesting that the induced PB1 specific CD8^+^ T-cell response provide markedly inferior protection compared to that induced by NP vaccination. To confirm this impression, groups of mice were vaccinated i.n. plus s.c. with one of the following adenovector constructs: AdIiPB1, AdNP or AdIiGP for control. Thirty days later these mice and a group of naïve controls were challenged i.n. with PR8 and 5 days later virus titers in the lungs were determined. As can be seen in Fig. 7, NP vaccination provided substantially better protection than vaccination against PB1, which still induced significant protection compared to naïve controls. Sham vaccination with an irrelevant vector did not induce any protection compared to naïve controls.

### Understanding the inferiority of PB1-specific CD8^+^ T cells compared to NP-specific T cells

As an extension of the above findings, we wanted to understand why vaccination using AdIiPB1 did not provide the same efficient protection as AdNP, despite seemingly high numbers of PB1-specific CD8^+^ T cells being generated following AdIiPB1 vaccination.

**Figure 8:**
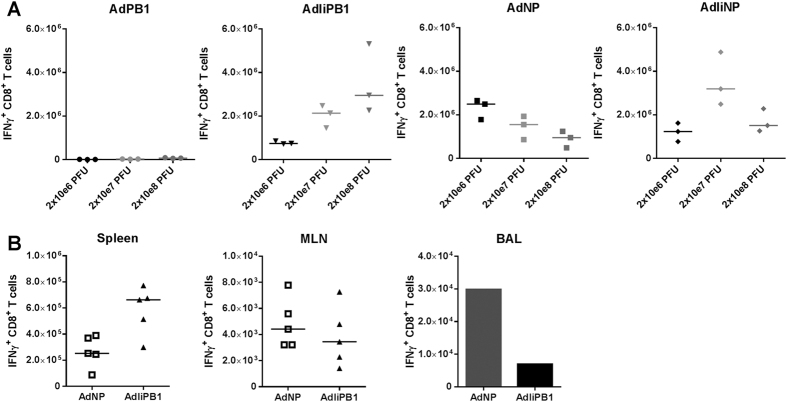
AdNP and AdIiPB1 induce similar number of CD8 T cells. (**A**) Groups of C57BL/6 mice were vaccinated s.c. with one of the doses, 10^6^, 10^7^ and 10^8^ PFU, of one of the following adenovectors: AdPB1, AdIiPB1, AdNP and AdIiNP. Fourteen days later numbers of antigen-specific CD8 T cells in the spleen were enumerated though flow analysis after stimulation with matching peptide. Each dot represents one animal and bars represent medians. (**B**) C57BL/6 mice were vaccinated with 2 × 10^7^ PFU AdIiPB1 or AdNP s.c. and i.n. and 30 days post vaccination spleen, MLN and BAL were isolated from animals. The number of IFN-γ^+^ CD8^+^ T cells in each organ site were determined though flow analysis after stimulation with matching peptide. Each dot represents one animal and bars represent medians (spleen and MLN). In BAL bars represent average number of IFN-γ^+^ CD8^+^ T cells per animal based on a pool from 5 animals.

As the first step in this analysis, we tested if the observed difference should reflect an intrinsic difference in the immunogenicity of the involved vaccines. To this end, we performed a dose/response experiment where groups of mice were vaccinated in the s.c. with one of the doses, 10^6^, 10^7^ and 10^8^ PFU, of one of the following adenovectors: AdPB1, AdIiPB1, AdNP and AdIiNP. Fourteen days later numbers of antigen-specific CD8 T cells in the spleen were enumerated. We found (Fig. 8A) that over a 100-fold dose range, three of the constructs (AdIiPB1, AdNP and AdIiNP) consistently induced substantial and almost equal numbers of antigen-specific CD8^+^ effector T cells (between 1–3 × 10^6^ antigen-specific cells), while even the highest dose of the fourth constructs (AdPB1) elicited very few cells (<10^5^/spleen). Besides underscoring the strong immunogenicity of the AdIiPB1 vector, these results confirmed that NP_396_-specific cells could be very effectively elicited even without tethering to Ii, whereas a PB1_703_-specific response of the same magnitude critically required tethering of this protein to Ii.

To further study the difference between PB1 and NP vaccination, we directly compared the numbers of PB1_703_ and NP_366_ -specific CD8^+^ T cells found in different organs (spleen, MLN and BAL) 30 days after s.c + i.n. vaccination with either AdIiPB1 or AdNP. Interestingly, in this situation AdIiPB1 vaccinated mice had more antigen-specific cells in the spleen compared to NP vaccinated mice, whereas similar numbers were found in MLNs, and more antigen-specific cells were recovered from the BAL of AdNP vaccinated animals (Fig. 8B). Thus, NP-specific cells seemed to accumulate near the airways to a higher degree than PB1- specific cells.

**Figure 9:**
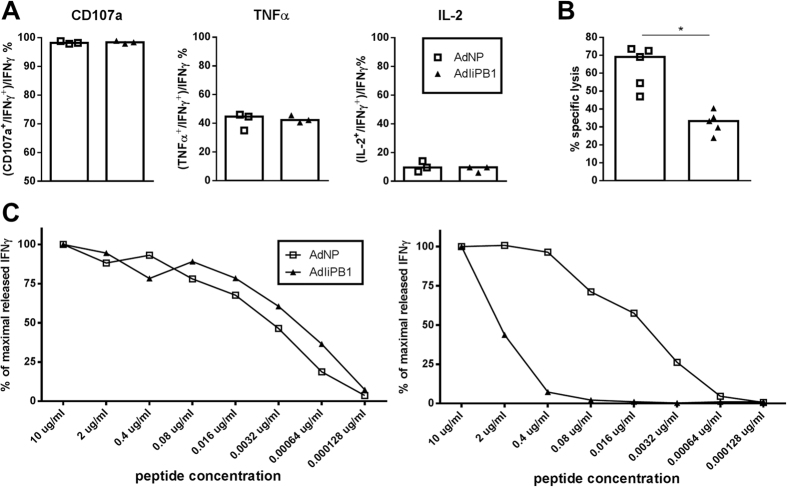
Comparison of T cells targeting PB1_703_ and NP_366_. (**A**) C57BL/6 mice were vaccinated with 2 × 10^7^ of PFU AdIiPB1 or AdNP s.c. and i.n. and 14 days post vaccination spleenocytes were isolated. The percentages of CD107a^+^, IL-2^+^ or TNFα^+^ cells out of the IFN-γ^+^ CD8^+^ T cells were determined though ICS and flow analysis after stimulation with matching peptide. (**B**) C57BL/6 mice were vaccinated with 2 × 10^7^ of PFU AdIiPB1 or AdNP s.c. and i.n. 30 days later mice were intravenously injected with a 1:1 mixed population of spleen cells loaded with PB1_703_ or NP_366_ under similar conditions. 16 hours after cell transfer, the fraction of injected cells recovered in the host spleens was determined by flow cytometry. (**C**) C57BL/6 mice were vaccinated with 2 × 10^7^ of PFU AdIiPB1 or AdNP s.c. and i.n. Fourteen days post vaccination splenocytes were isolated from 3 mice and pooled. The fraction of CD8^+^ T cells producing IFN-γ as a function of the peptide concentration used for loading of stimulator cells (functional avidity) was determined though flow analysis after 5 hours of co-incubation with peptide-loaded splenocytes (left hand side). To study the temporal stability of surface MHC I-peptide complexes effector cells were also added 6 hours later, and again the fraction of CD8^+^ T cells producing IFN-γ as a function of the peptide concentration used to load the stimulators cells were determined though flow analysis after 5 hours of co-incubation (right hand side).

Next, we made a head-to-head comparison of the quality of adenovirus vector elicited PB1_703_- and NP_366_-specific CD8^+^ T cells using several parameters as end-points for our analysis. To evaluate effector cell status/functionality, PB1- and NP-specific CD8^+^ T cells were optimally stimulated with peptide for 5 hours, and the fraction of IFNγ producing cells also degranulating or co-producing TNF-α or IL-2 was determined. As can be seen in Fig. 9A, a similar composition regarding functional subsets was observed irrespective of the antigenic specificity of the CD8^+^ T cells.

Furthermore, we wanted to compare the capacity of PB1- and NP-specific cells to recognize their relevant targets *in vivo*. As a functional parameter to evaluate T-cell recognition *in vivo*, we performed standard *in vivo* cytotoxicity assays in which the cytolytic capacity is determined by the ability to find and kill injected peptide loaded spleen cells^18^. Thirty days after vaccination with AdIiPB1 or AdNP, these mice and naïve controls received a 1:1 mixed population of spleen cells loaded with PB1_703_ or NP_366_ under similar conditions. Sixteen hours after cell transfer, the fraction of injected cells recovered in the host spleens was determined by flow cytometry. As can be seen in Fig. 9B, levels of *in vivo* killing did not match the frequencies of relevant antigen-specific CD8^+^ T cells in each type of host, as a significantly lower killing of the injected cells was observed in AdIiPB1 vaccinated mice. Since the frequency of relevant effector cells in spleen is higher in AdIiPB1 vaccinated mice compared to AdNP vaccinated mice, and the quality of the effector cells apparently does not differ, this result suggests that there is reduced *in vivo* recognition of the relevant target cells in AdIiPB1 vaccinated mice.

Seeking an explanation to this conundrum, we compared the functional avidity of PB1 and NP specific CD8^+^ T cells, i.e. the ability of these antigen specific cells to sense peptide presentation. To this end, we determined the relative frequencies of IFN-γ producing CD8^+^ T cells induced by *ex vivo* incubation in the presence of decreasing concentrations of either peptide. Interestingly, the threshold for activation of the PB1_703_- and NP_366_-specific CD8^+^ T-cell subset was similar under these conditions (data not shown). Therefore, in order to be able to explain the results of the *in vivo* cytotoxic analysis, we hypothesized that the peptide/MHC class I complexes were less stable with the PB1 peptide and that with time, this would significantly limit the triggering of PB1-specific CD8^+^ T cells, thus explaining the observed difference in a long-term assay like the *in vivo* cytotoxic test. To evaluate this possibility, an experiment similar to that just described above was set up with the notable difference that instead of peptide in solution, washed naïve splenocytes loaded with decreasing concentrations of either peptide were used for stimulation of IFNγ production. Either immediately or 6 hours later, splenocytes from vaccinated mice were added and intracellular staining was performed after 5 hours of co-incubation according to standard procedures. A comparison of the results obtained under these conditions (Fig. 9C) revealed a clear difference in the presentation of the two peptides, as PB1_703_-specific CD8^+^ T cells required a much higher loading concentration of peptide to be fully stimulated when the addition of responder T cells was delayed. From this, we conclude that the functional avidities of PB1_703_ and NP_366_-specific CD8^+^ T cells are very similar, but the PB1_703_ peptide is less stably expressed on the cell surface than the NP_366_ peptide.

## Discussion

Influenza remains one of the most widespread human infections despite annual vaccination programs. The virus continuously changes through antigenic drift and shift creating new seasonal and pandemic strains. As previously mentioned, the current vaccines are unable to provide heterosubtypic protection and there is a need for development of a new vaccine strategy against influenza A infections. This was especially highlighted in the recent H1N1 2009 pandemic as well as in the increased number of cases and deaths of the highly pathogenic avian influenza in humans, H5N1, in the last year^19^. Here, we have investigated an adenovirus encoding the internal influenza protein PB1 as a potential vaccine candidate. PB1 is highly conserved and expressed by all influenza A viruses making it a good target for a broad protection^8^. AdPB1 does not at first glance seem to show much promise, but by adding invariant chain to the antigen, we observe a very marked increase in the number of PB1-specific CD8^+^ T cells induced. These results confirm previous studies, where the addition of invariant chain has been found to increase CD8^+^ T cell responses. This is especially true for weaker antigens containing subdominant epitopes^15^,^20^. The mechanism behind this phenomenon is, however, not yet fully elucidated as Ii normally is involved in MHC II presentation, but this does not appear to play a role in this case.

Even though AdIiPB1 could induce a high number of PB1-specific CD8^+^ T cells, we did not detect significant protection when AdIiPB1 was used in s.c. vaccination. In our previous study with AdNP, we saw an increase in the number of CD8^+^ T cells in the lungs and MLN when vaccinating both s.c. and i.n. As with AdNP, combined local and systemic vaccination with AdIiPB1 resulted in higher numbers of CD8^+^ T cells in the MLN and BAL, and this correlated with a reduction in viral titers in the lungs 5 and 7 days post infection. Mice vaccinated only i.n. had higher mortality, lost more weight and eventually had slightly higher viral titers compared to mice vaccinated both i.n. and s.c. Based on these findings and on our previous experience with AdNP vaccination, we speculate that a good systemic priming of T cells is important for a more prolonged T-cell response, which explain why these mice are the last to lose weight. A similar mechanism has been discussed concerning tuberculosis infection, where a combination of i.n. and s.c. immunization gives the benefit of both a good early and a late response^21^.

Interestingly, PB1-specific T cells do not seem to be nearly as efficient in controlling influenza replication as NP-specific cells. There may be several reasons for that. First, vaccine-driven NP-specific CD8^+^ T cells tend to accumulate in MLN and BAL to a higher degree than similarly stimulated PB1-specific T cells, at least early in the memory phase. The underlying mechanism for this is not clear, but following primary influenza infection the expansion of NP-specific cells is prolonged and contraction delayed. This is due to continued presentation of NP by a subset of lymph node DCs that are CD103^−^CD11b^high^ and this programs these T cells for a robust recall response^22^. If antigen presentation is similarly biased after adenovector immunization, which is known to cause sustained local antigen presentation^23^, this may lead to a preferential accumulation of NP-specific cells in the airways compared to PB1-specific cells. Given that a more diverse range of APCs present NP^24^ local accumulation is very likely to increase further following flu challenge. This difference and in particular migratory CD103^+^ DCs have been found to induce an early and efficient presentation of NP in the MLN that favors the expansion of CD8^+^ T cells with this specificity above others^25^. Additionally, we find that under similar conditions, PB1-specific T cells are less efficient in recognizing and eliminating their relevant target cells *in vivo*, probably because of a lower stability of the targeted peptide/MHC combination. Since low peptide/MHC complex stability will tend to reduce epitope expression also on infected target cells, this, combined with the reduced numbers of PB1-specific CD8^+^ T cells present locally, might explain why PB1-specific cells provide less efficient protection compared to NP-specific CD8^+^ T cells.

What are the implications for human vaccination against influenza A? First, our results underscore that a potent systemic response does not guarantee efficient protection; not surprising localization, but also fine specificity of the involved cells matter more. Second, even though our results are based on analysis of only two dominant epitopes in a single inbred mouse strain, these and other results^22^,^24^ suggest that relevant local expression of PB1 in the lungs is limited compared to that of NP. If this observation can be extrapolated to humans, local accumulation as well as the efficiency of PB1-specific effector CD8^+^ T cells would be less substantial also in human patients.

Thus, the protection from AdIiPB1 vaccination is not complete. However, compared to real life human infection, the dose of influenza used for challenge in our animal model is probably much higher than what humans are normally exposed to. Furthermore, PB1 induced immunity may matter, if immunization against this antigen is combined with other adenovirus encoded flu antigens. Adding PB1 will then increase the breadth of the induced T-cell response and might contribute significantly to protection if some of the other responses fail due to mutations in the involved epitopes. Hence, PB1 could still represent a relevant vaccine target to consider in a future flu vaccine.

## Materials and Methods

### Mice

Female C57BL/6 mice (H-2^b^), 6–8 weeks old, were obtained from Taconic Farms (Ry, Denmark) and housed in a specific pathogen–free facility. Upon arrival, all mice were allowed to acclimatize for ≥1 week at the facility before being used in experiments. Experiments were conducted in accordance with national Danish guidelines (Amendment # 1306 of November 23, 2007) regarding animal experiments as approved by the Danish Animal Experiments Inspectorate, Ministry of Justice, permission numbers 2015-15-0201-00623, and the mice were housed in an AAALAC accredited facility in accordance with good animal practice as defined by FELASA.

### Adenoviral vectors

A replication-deficient E1-deleted Adenovirus serotype 5 vector with a nonfunctional E3 gene, expressing the PB1 gene from influenza strain A/Puerto Rico/8/34 unlinked or linked to invariant chain (designated AdPB1 or AdliPB1) was produced as described previously^26^. Adenoviral particles were purified using standard methods, aliquoted, and frozen at −80 °C in 10% glycerol. Insert was verified by sequencing (data not shown). Infectivity of the adenovirus stocks was determined using Adeno-X Rapid Titer Kit (Clontech Laboratories, Mountain View, CA). The production of other adenovectors used here has previously been described^9^.

### Vaccination

Vaccination was given subcutaneous (s.c.) and/or intranasal (i.n.). Mice s.c. vaccinated were briefly anesthetized with isoflurane and injected in the right foot pad with 2 × 10^7^ particle forming units (PFU) in 30 μl of PBS. Mice vaccinated i.n were first anaesthetized by intraperitoneal (i.p.) injection with avertin (2,2,2 tribromoethanol in 2-methyl-2-butanol, 250 mg/kg) and then vaccinated with 2 × 10^7^ PFU in 30 μl of PBS in the nostrils.

### Virus challenge

Challenge was performed with diluted A/Puerto Rico/8/34 (PR8) influenza virus. The lethal dose was determined, and 1–3 LD_50_ were used for all challenge. Mice to be challenged were first anaesthetized by i.p. injection with avertin (2,2,2 tribromoethanol in 2-methyl-2-butanol, 250 mg/kg) and subsequently infected i.n. with 30 μl of appropriately diluted influenza virus. After influenza infection, animals were weighed daily and euthanized by cervical dislocation if the weight loss exceeded 25% or the experiment was terminated 21 days post challenge.

### Single cell preparation and flow cytometry

To obtain single cells from spleens and mediastinal lymph node (MLN), these organs were aseptically isolated and pressed through a fine steel mesh. For brochoalveolar lavage (BAL) sampling, mice were first anaesthetized using avertin and exsanguinated in order to reduce the risk of lymphocyte contamination from the blood. Next, the trachea was exposed and a small incision was made. A venflon was inserted into the incision and the lungs were flushed 3 times with ice-cold Hanks BSS medium. BAL samples were pooled within groups in order to obtain enough cells for analysis. All samples were centrifuged and re-suspended in Hanks BSS. Cells were then counted on an automated cell counter, Countess (Invitrogen). This was followed by centrifugation and resuspension in RPMI 1640 cell culture medium containing 10% FCS supplemented with 2-ME, l-glutamine, and penicillin-streptomycin. For enumeration of Ag-specific T cells, splenocytes were incubated at 37 °C and 5% CO_2_ for 5 h in the presence of 1 μg/ml PB1_703_ peptide (SSYRRPVGI) or NP_366_ peptide (ASNENMETM), 50 IU/ml IL-2, and 3 μM monensin; cells incubated without peptide or with irrelevant peptide was included for control. Cells were then stained for surface markers. Subsequent to surface staining, the cells were washed, permeabilized, and stained for intracellular cytokines^10^. Samples were analyzed using a LSR II (BD Biosciences), and data analysis was conducted using FlowJo v7.6.5 software (TreeStar); see Supplementary Figure 1 for our gating strategy.

### Antibodies for flow cytometry

All antibodies were purchased from Nordic Biosite. The following flourochrome-conjugated monoclonal rat anti-mouse antibodies were used for surface and intracellular cytokine staining: PerCP-Cy5.5 conjugated α-CD8 (clone 53-6.7), FITC conjugated α-CD44 (clone IM7), APC conjugated α-IFNγ (clone XMG1.2), APC/Cy7 conjugated CD44 (clone IM7), PE conjugated IL-2 (clone JES6-5H4), PE/Cy7 conjugated TNFα (clone MP6-XY22) and AlexaFlour488 conjugated CD107a (clone 1D4B).

### Influenza virus plaque assay

Lungs were homogenized using sterilized sand and a mortar and pistil, 1% FBS in PBS was added to obtain a 10% weight/volume suspension. Samples were spun down at 600 G for 15 min at 4 °C, and the supernatant was transferred to a new tube and kept on ice until use.

Madin-Darby Canine Kidney epithelial (MDCK) cells were used for influenza plaque assay and grown in complete medium. 4.5 × 10^4^ MDCK cells in 100 μl medium were grown in 96-well plates overnight. For the plaque assay, 10-fold dilutions of the lung suspensions were prepared using an influenza growth medium containing DMEM 1965 medium with 2 mM L-glutamine, 200 IU/ml penicillin, 50 μg/ml streptomycin, 0.2% BSA, 1% sodium-pyruvate and 5 units/ml TPCK Trypsin. MDCK-cells were first washed twice with PBS and then incubated with 50 μl of virus dilution for 2 hour at 37 °C, 5% CO2. Samples were then removed, and an overlay medium containing 2x minimum essential medium (MEM) eagle supplemented with 0.4% BSA, 10% NaHCO3, 2% Streptomycin, 2% penicillin and 5 units/ml TPCK trypsin mixed 1:1 with 1.8% methylcellulose was added to the cells. Cells were incubated for 48 hour at 37 °C, 5% CO2. Then overlay was removed and wells were washed 2x with PBS. Cells were fixated with 4% formaldehyde in PBS for 30 min, RT. After fixation cells were washed twice with PBS and permeabilized with warm 0.5% Triton-X in Hanks balanced salt solution medium for 10 min, RT. Cells were subsequently washed twice with PBS. Next, cells were incubated with primary α-influenza nucleocapsid A mAb (Nordic Biosite) diluted 1:1500 in 10% FBS in PBS for 1 hour at 37 °C, 5% CO2. Antibody was removed and cells washed 5x. This was followed by incubation with a secondary goat α-mouse HRP conjugated mAb (Dako) diluted 1:500 in 10% FBS in PBS for 1 hour at 37 °C, 5% CO2. After secondary antibody incubation cells were washed 5x with PBS. 200 μl substrate solution containing 3 mg/ml 3-amino-9-ethylcarbazole and 0.07% H_2_O_2_ and 5 mM citrate phosphate buffer pH5 was added to wells for 30 minutes, RT. Substrate was removed and cells were washed once with PBS before counting. All samples were run in duplicates. Plaque forming units per g lung tissue were calculated accordingly:


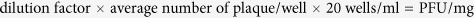


Detection level was calculated to be 1000 PFU/mg, corresponding to 3.0 log PFU/mg

### *In vivo* cytotoxicity assay

Splenocytes from naive B6.SJL mice (CD45.1 positive) were pulsed with NP_366_ or PB1_703_ (10 μg/spleen) for 30 minutes at 37 °C. After incubation with peptide, the cells were washed and stained with 0.2 μM CFSE (NP_366_) or 2 μM (PB1_703_). Following another washing step, the labeled cells were mixed in a 1:1 ratio, and 2 × 10^7^ cells were injected i.v. into vaccinated C57BL/6 mice (CD45.1 negative); 16 h later, recipient splenocytes were isolated, and target cells were identified using flow cytometry by the expression of CD45.1 and CFSE staining. The percentage of killing was calculated using the following equation: 100−[(percentage of Influenza peptide-labeled cells in infected mice/percentage of cells labeled with irrelevant peptide in infected mice)/(percentage of Influenza peptide-labeled cells in uninfected mice/percentage of cells labeled with irrelevant peptide in uninfected mice) ×100] as described in ref. 18.

### Statistical analysis

Analysis was performed using Graphpad Prism software v6.01. Statistical comparison of two groups was done using the Mann-Whitney test (two-tailed). Data from all experiments with more than two groups were first compared using a one-way ANOVA test, and, only if groups were found to differ significantly, this analysis was followed by pair-wise comparisons using Mann-Whitney rank sum test. A significant difference between two groups was acknowledged if p < 0.05. Statistical significance was marked with an asterix (*).

## Additional Information

**How to cite this article**: Uddbäck, I. E. M. *et al*. PB1 as a potential target for increasing the breadth of T-cell mediated immunity to Influenza A. *Sci. Rep*. **6**, 35033; doi: 10.1038/srep35033 (2016).

## Supplementary Material

Supplementary Information
