# Supplementary material for: PB1 as a potential target for increasing the breadth of T-cell mediated immunity to Influenza A
Source: Sci Rep. 2016 Oct 7;6:35033. doi: 10.1038/srep35033 (PMC5054373; doi:10.1038/srep35033)

PB1 as a potential target for increasing the breadth of T-cell mediated immunity to Influenza A

Ida E M Uddbäck, Maria A Steffensen, Sara R Pedersen, Loulieta Nazerai, Allan R Thomsen, Jan P Christensen*

Department of Immunology and Microbiology,
University of Copenhagen,
Copenhagen, Denmark

Supplementary figure 1. Gating strategy

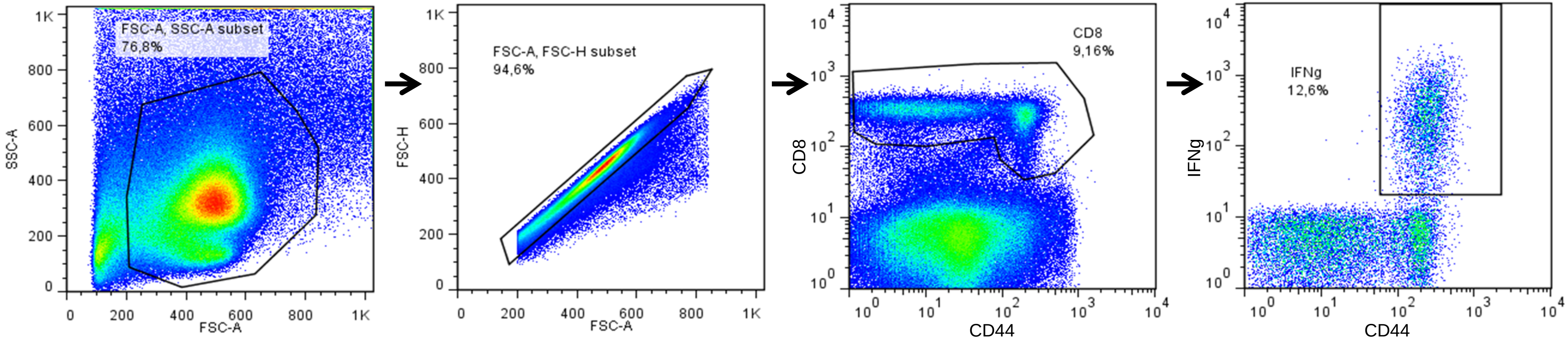

Supplement: Supplementary Information [file srep35033-s1.pdf]
